# Fibromyalgia as a Confounder of Disease Activity Assessment in Inflammatory Arthritis

**DOI:** 10.5152/ArchRheumatol.2026.26327

**Published:** 2026-05-11

**Authors:** Mete Kara

**Affiliations:** Department of Rheumatology, İzmir City Hospital, İzmir, Türkiye

To the Editor,

We read with great interest the recent article by Caglayan et al^^1^^ evaluating the coexistence of fibromyalgia (FM) in patients with rheumatoid arthritis (RA) and spondyloarthritis, and its relationship with disease activity and treatment status, representing an important clinical issue that frequently complicates disease assessment in inflammatory rheumatic diseases.

Fibromyalgia is a common comorbidity in inflammatory arthritis and represents a major challenge in routine clinical practice. The overlap between inflammatory pain and centrally mediated pain mechanisms complicates the interpretation of disease activity measures. Several studies have demonstrated that FM substantially amplifies patient-reported components of composite disease activity indices, inflating scores independent of objective markers such as C-reactive protein (CRP) and swollen joint count.^2^^,^^3^ Consequently, elevated scores in patients with concomitant FM may not accurately reflect active inflammation.

Consistent findings have been reported across several independent cohorts, including our previously published study.^4^ Pollard et al^^2^^ showed that fibromyalgic RA patients had significantly higher disease activity score (DAS)-28 scores, with an odds ratio of 14.3 for being classified as having active disease. Duffield et al,^^3^^ in a meta-analysis of 40 studies, confirmed that concomitant FM was associated with a mean DAS28 increase of 1.24 points in RA and 2.22 points on the Bath Ankylosing Spondylitis Disease Activity Index (BASDAI) in ankylosing spondylitis, with objective markers remaining unaffected. Salaffi et al^^5^^ similarly reported FM prevalence of 14.9% in axial spondyloarthritis, with significantly higher Ankylosing Spondylitis Disease Activity Score and BASDAI scores in FM-comorbid patients. In our study of 478 patients including 398 with RA and 80 with psoriatic arthritis (PsA), FM prevalence was 19.3% in RA and 18.7% in PsA with significantly higher DAS28-CRP (4.48 vs 3.51, *P* = .005) and Disease Activity Index for Psoriatic Arthritis (26 vs 14, *P* < .001) scores in FM-comorbid patients,^4^ supporting the concept that FM modifies disease activity assessment rather than indicating uncontrolled inflammation.

Regarding biologic or targeted synthetic disease-modifying antirheumatic drugs (b/tsDMARD) use, an interesting discrepancy emerges between studies. While our study observed higher bDMARD use among PsA patients with concomitant FM (*P* < .001), the current study reports higher use in RA patients without FM (*P* = .030).^1^ This difference may reflect variations in FM recognition, disease duration, or local treatment protocols. Such variability may also stem from differences in FM diagnostic criteria applied and the single-center retrospective design inherent to both studies,^3^ and cross-study comparisons should therefore be interpreted cautiously.

This issue has direct implications for treatment evaluation. In patients with concomitant FM, persistently high disease activity indices may be interpreted as inadequate therapeutic response, potentially leading to unnecessary treatment escalation or premature switching of b/tsDMARD.

The study by Caglayan et al^^1^^ contributes meaningfully to the literature emphasizing the importance of distinguishing inflammatory activity from centrally mediated pain. Growing evidence suggests that FM may substantially influence composite disease activity indices in inflammatory arthritis, and clinicians should remain aware of its potential confounding effect when interpreting DASs and making treatment decisions.

## Data Availability Statement:

The data that support the findings of this study are available on request from the corresponding author.

## Artificial Intelligence Usage Statement:

The author declared that no artificial intelligence tool was used in the preparation of the manuscript.

## Peer-review:

Externally peer-reviewed.

## Author Contributions:

Concept – M.K.; Analysis and/or Interpretation – M.K.; Literature Search – M.K.; Writing – M.K.; Critical Review – M.K.

## Declaration of Interests:

The author has no conflicts of interest to declare.

## Funding:

The author declare that this study received no financial support.
